# Male sexual health predictors during the Covid-19 outbreak: a multicenter study

**DOI:** 10.1186/s12301-022-00318-2

**Published:** 2022-09-24

**Authors:** Diaa-Eldin Taha, Ali Ibrahim, Samer El-Halwagy, M. A. Elbaset, Rawdy Ashour, Hossam Nabeeh, Ibrahem Ismail Samaha

**Affiliations:** 1 Department of Urology, Faculty of Medicine, Kafrelsheikh University, Kafrelsheikh, Egypt; 2 Department of Urology, Zagazig University, Zagazig, Egypt; 3 Department of Urology, Urology and Nephrology Center, Mansoura University, Mansoura, Egypt

**Keywords:** COVID-19, Depressive disorder, Pandemics, Sexual health

## Abstract

**Background:**

Pandemic-induced feelings of fear and worry are all psychological implications of the COVID-19 pandemic. The goal of this study was to see how the COVID-19 pandemic affected male Sexual Health and to look for plausible predictors.

**Methods:**

Married males were asked to fill out an Arabic Sexual Health questionnaire. Before and during the lockdown. Additionally, generalized Anxiety Disorder-7 and International Index of Erectile Function-5 questionnaires.

**Results:**

A multicenter study. The survey was completed by 281 men in total. Only 130 males (47.3%) were satisfied with their Sexual performance before lockdown, compared to 170 males (56.5%) who were not satisfied (P 0.000). Financial issues (*P* ≤ 0.000), smoking habit prior to lockdown (*P* ≤ 0.001), spots practice (*P* ≤ 0.001), smoking during lockdown (*P* ≤ 0.001), presence of depressive disorder on the PHQ-9 total score (*P* ≤ 0.001), diagnosis of anxiety on the GAD-7 score (*P* ≤ 0.001), and presence of ED on the IIEf-5 questionnaire (*P* ≤ 0.001) were all found to be significant on univariate analysis. On bivariate analysis, financial issues (odds ratio [OR]: 3.56, *P* ≤ 0.000), presence of anxiety on GAD-7 (OR: 6.40, *P* ≤ 0.001), PHQ score (OR: 2.50, *P* ≤ 0.001), and diagnosis of ED on the IIEF-5 scale (OR: 7.50, *P* ≤ 0.001) were significantly associated with Sexual relationship stress and Sexual Health.

**Conclusion:**

During and after COVID-19 lockdown, the presence of anxiety on the GAD-7 scale, PHQ score, and the diagnosis of ED on the IIEF-5 scale were all independent predictors of Sexual Health.

## Background

The novel severe acute respiratory syndrome coronavirus 2 (SARS-CoV-2) has spread over the world since its discovery in Wuhan, China, and the World Health Organization labeled the consecutive coronavirus disease 2019 (COVID-19) a pandemic shortly after [1].

The first incidence was reported in Egypt in February 2020. As a result, the number of cases has been increasing, with a death rate of 4.8 percent observed [2]. Egypt enacted a lockdown starting in mid-March 2020, closing all non-essential enterprises (including schools), and all employees and students worked from home [2].

COVID-19 severity and mortality were shown to be higher in males over the world. Male predominance was attributed to delayed viral RNA clearance, sex-related immune response differences, and hormonal milieu abnormalities, according to researchers [3].

Fear, worry, anxiety, and concern were expressed by specific populations, including older folks, health care workers, and people with chronic disorders, as a result of the virus's rapid global spread and ambiguity about treatment and disease outcome [2]. This pandemic also resulted in life-threatening situations, unemployment, reduced income, and family or partner separation [4].

Females have been observed to experience more psychological anguish as a result of COVID-19 [5]. The extraordinary pandemic morbidity, mortality, and lockdown measures are predicted to have an impact on the population's mental health, as well as potential changes in Sexual Health practices [6].

COVID-19 pandemic was linked to poorer Sexual Health in both genders, according to a comprehensive study conducted in Egypt. Females, on the other hand, had higher levels of worry and despair, putting them at a higher risk of Sexual Health problems and dissatisfaction [7].

We expected that because Egyptian males are more vulnerable to psychological stress, they would experience more Sexual Health tension. We conducted a multicenter study to investigate the impact of the COVID-19 pandemic on male Sexual Health in Egypt and to assess potential risk factors.

## Methods

It is a multicenter study involving three tertiary referral centers. Of which two were a Covid-19 isolation centers. Data were obtained online due to COVID-19-related constraints on face-to-face recruitment. We launched an online page at the end of April 2020 to engage with participants and improve study recruitment. Google Forms was used to administer the survey online. If possible, some patients completed the questionnaire during hospital visits. Ethical approval number was taken from university ethical approval committee.

### Structure of the questionnaire

A participant information document, a consent form, and a debrief sheet were all included in the questionnaire. Participants provided demographic and clinical information. Participants' Sexual Health satisfaction was assessed by inquiring if they were satisfied with their Sexual Health before and after lockdown.

### Assessment of depressive symptoms

The study participants were given the Arabic validated version of the Patient Health Questionnaire (PHQ-9) [8]. The PHQ is an effective screening tool for depressive disorder and other mental illnesses that are widely seen in primary care settings. The PHQ-9 is a 9-item depressive disorder module from the entire PHQ, with each item ranging from 0 (not at all) to 3 (very) (nearly every day) [9]. It is based on diagnostic criteria from the Diagnostic and Statistical Manual of Mental Disorders (DSM-IV), allowing the patient's mood to be measured in the weeks leading up to the consultation. PHQ-9 scores range from 0 to 27, with 0 being the lowest and 27 being the highest [9]. The PHQ-9 cut points were minimum or none (four); mild or minimal (five to nine); moderate (10–14); moderately severe (15–19); and severe depressive disorder (20). (20–27) [9].

### Assessment of anxiety

The Arabic version of the Generalized Anxiety Disorder-7 (GAD-7) questionnaire was utilized in this study [8]. During the lockdown time, the individuals' generalized anxiety ratings were assessed. GAD-7 is a self-reporting questionnaire that takes around 2 min to complete and uses DSM-IV criteria for generalized anxiety disorder to measure an individual's anxiety state. Each of the seven items is graded on a four-point Likert scale based on the frequency with which the symptom has occurred. Total scores vary from 0 to 21, with item scores ranging from 0 to 3 (almost every day). The GAD-7 scores were interpreted as follows: Score 4 indicates no anxiety, 5–9 indicates mild anxiety, 10–14 indicates considerable anxiety, and 15 indicates severe anxiety [10].

### Evaluation of male sexual health function

Erectile function was assessed using the Arabic version of the 5-item International Index of Erectile Function (IIEF-5) questionnaire. It's a 5-point scale that rates maintenance ability, erection confidence, maintenance frequency, erection strength, and intercourse satisfaction on a range of 1 to 5. Based on the scores, erectile dysfunction was divided into five categories: severe (5–7), moderate (8–11), mild to moderate (12–16), mild (17–21), and no erectile dysfunction (22–25) [11]. Male and female Sexual Health satisfaction are assessed. The Arabic validated version of the Index of Sexual Satisfaction (ISS), which consists of 25 items on a 7-grade scale, was used [12]. The ISS is used to examine various elements of Sexual pleasure, including contentment with Sexual life, Sexual emotion expression toward partners, Sexual partnership quality, and reasons for Sexual intercourse. The final ISS score is calculated by adding item points and applying a specific ISS formula. The overall score ranges from 0 (complete satisfaction) to 100 (complete dissatisfaction) (minimal satisfaction). Higher scores imply a poor quality of Sexual Health and stress in the marital relationship's Sexual Health. At a cutoff point of 30, clinically severe Sexual Health dysfunctions are detected [13].

### Sample size calculation

The sample size was determined based on the maximum numbers of participants that have the potential to participate. All participants were consented prior to enrollment in the study. Participants enrolled in the study were anonymous to the data analysis process.

### Statistical analysis

SPSS 21.0 for Windows was used to analyze the data (SPSS, USA). The distributions of numeric variables were evaluated using normality tests (Kolmogorov–Smirnov test). If the distribution of numeric variables were normal, statistical analysis was performed using parametric Student’s t-tests. To examine numerical variables with a skewed distribution, Mann–Whitney U-tests were performed. The chi-squared or Fisher's exact tests were used to examine categorical variables. The level of statistical significance was set at 5% (P < 0.05). A highly significant difference was present if *P* ≤ 0.001.

## Results

Between April 30, 2020, and June 30, 2021, a total of 281 males were enrolled. The average (SD) age was 45.4 ± 14.5 years. The marriages lasted anywhere from one to twenty-six years. No patients in our group were found to be COVID-19 positive or had to be quarantined. There were 88 men (39.2%) who had chronic diseases. The most common related illness: diabetes mellitus (19.7%). The clinical and demographic features of the study participants are summarized in Table 1.

**Table 1:** Correlation of sexual satisfaction during covid outbreak

	Sexual satisfaction post covid				P
	Very strong	Strong	Weak	No desire	
Age (mean ± SD)	45.4 ± 14.5				0.043
Weight (mean ± SD)	74.8 ± 8.54				0.00
Height (mean ± SD)	175.33 ± 4.40				0.04
Marriage duration (mean ± SD)	18.75 ± 10.0				0.01
Coitus pre covid per week (mean ± SD)	3.23 ± 1.12				0.00
Coitus post covid per week(mean ± SD)	5.35 ± 1.32				0.00
Financial problems during lockdown (yes) No %	28 (23.7%)	43 (36.4%)	36 (30.5%)	11 (9.3%)	0.07
Smoking pre covid (yes) No %	31 (17.3%)	78 (43.6%)	46 (25.7%)	24 (13.4%)	0.13
Smoking post covid (higher) N0%	11 (23.9%)	23 (50.0%)	12 (26.1%)	0 (0%)	0.000
Sports practice post Covid (yes) No %	15 (28.8%)	25 (48.1%)	9 (17.3%)	3 (5.8%)	0.008
DM No %	2 (3.6%)	27 (48.2%)	11 (19.6%)	16 (28.6%)	0.000
HTN No %	3 (13.0%)	13 (56.5%)	7 (30.4%)	0 (0.0%)	0.000
IHD No %	1 (9.1%)	4 (36.4%)	0 (0%)	6 (54.4%)	0.000
ED pre covid No %	0 (0.0%)	14 (36.8%)	18 (47.4%)	6 (15.8%)	0.000
Work from home No %	24 (23.1%)	42 (40.4%)	30 (28.8%)	8 (7.7%)	0.10
*Sexual practice post covid*					
Perfect	12 (36.4%)	14 (42.4%)	5 (15.2%)	2 (6.1%)	0.00
Good	14 (20.0%)	34 (48.6%)	16 (22.9%)	6 (8.6%)	
Average	12 (44.4%)	13 (48.1%)	1 (3.7%)	1 (3.7%)	
Poor	10 (6.7%)	52 (34.9%)	62 (41.6%)	25 (16.8%)	
*Sexual practice pre covid*					
Perfect	9 (18.4%)	20 (40.8%)	14 (28.6%)	6 (12.2%)	0.02
Good	22 (25.6%)	38 (44.2%)	19 (22.1%)	7 (8.1%)	
Average	8 (10.5%)	28 (36.8%)	33 (4.3%)	7 (9.2%)	
Poor	9 (13.2%)	27 (39.7%)	18 (26.5%)	14 (20.6%)	
*Sexual desire pre covid*					
Average	2 (3.0%)	26 (38.8%)	38 (56.7%)	1 (1.5%)	0.00
Good	17 (22.4%)	36 (47.4%)	19 (25.0%)	4 (5.3%)	
Great	29 (45.3%)	25 (39.1%)	2 (3.1%)	8 (12.5%)	
Bad	0 (0.0%)	26 (36.1%)	25 (34.7%)	21 (29.2%)	
*Sexual desire post covid*					
Average	15 (14.3%)	61 (58.1%)	21 (20.0%)	8 (7.6%)	0.000
Good	0 (0.0%)	21 (34.4%)	38 (62.3%)	2 (3.3%)	
Great	33 (63.5%)	9 (17.3%)	6 (11.5%)	4 (7.7%)	
Bad	0 (0.0%)	22 (42.3%)	15 (28.8%)	15 (28.8%)	
*Sexual satisfaction pre covid*					
Very strong	45 (58.4%)	21 (27.3%)	7 (9.1%)	4 (5.2%)	0.00
Strong	3 (3.3%)	47 (51.6%)	33 (36.3%)	8 (8.8%)	
Weak	0 (0.0%)	43 (39.4%)	44 (40.4%)	22 (20.2%)	
*Wife harassment precovid*					
Always	42 (57.5%)	19 (26.0%)	10 (13.7%)	2 (2.7%)	0.000
Unusual	6 (5.7%)	54 (51.4%)	35 (33.3%)	10 (9.5%)	
Never	0 (0.0%)	32 (34.8%)	39 (42.4%)	21 (22.8%)	
Sometimes	0 (0.0%)	6 (85.7%)	0 (0.0%)	1 (14.3%)	
*Wife harassment post covid*					
Always	36 (59.0%)	19 (31.1%)	4 (6.6%)	2 (3.3%)	0.00
Usual	11 (9.9%)	58 (52.3%)	32 (28.8%)	10 (9.0%)	
Never	0 (0.0%)	26 (27.4%)	48 (50.5%)	21 (22.1%)	
Sometimes	1 (8.3%)	10 (83.3%)	0 (0.0%)	1 (8.3%)	
PDEi post covid	8 (8.7%)	45 (48.9%)	22 (23.9%)	17 (18.5%)	0.003

Only 130 males (47.3%) said they were satisfied with their Sexual Health before COVID-19 lockdown, compared to 170 males (56.5%) who said they were content throughout the lockdown period (*P* = 0.000). During the lockout, 118 (42%) of the participants had financial problems. A total of 105 people (37.4%) increased their smoking habits.

According to the reported PHQ-9 scores, 64.4% of males assessed as no depressive disorder on the PHQ-9 scale had no or minimal depressive disorder. Males had a higher mean depressive disorder score (7.4 ± 2.1) (*P* = 0.001). On the GAD-7 questionnaire, more than half of the men (67.6%) answered "no anxiety." Males had a higher mean anxiety GAD-7 score (5.3 ± 2) (*P* = 0.001).

On the one hand, 191 male individuals (68.0%) had no ED scores on the IIEF-5 questionnaire prior to the COVID-19 lockdown, while 54 patients (18.3%) had mild ED and 35 (12.7%) had moderate ED. The IIEF-5 score was 21.9 ± 3.2 on average. During the COVID-19 lockout, however, 176 male patients (62.2%) had no ED ratings on the IIEF-5 questionnaire, 64 individuals (22.3%) had mild ED, and 55 (15.1%) had strong ED. The IIEF-5 score was 19.9 ± 4.2 on average.

Financial issues (*P* = 0.000), smoking habits prior to lockdown (*P* = 0.001), spots practice (*P* = 0.001), smoking during lockdown (*P* = 0.001), presence of depressive disorder on the PHQ-9 total score (*P* = 0.001), diagnosis of anxiety on the GAD-7 score (*P* = 0.001), and presence of ED on the IIEf-5 questionnaire (*P* = 0.001) were all significantly related to the ISS total score. The ISS total score had no significant relationship with residence (*P* = 0.407), marriage duration (*P* = 0.057), or chronic illnesses (*P* = 0.466).

After controlling for other factors, the results of a linear regression analysis of factors affecting ISS score in males revealed that financial issues (odds ratio [OR]: 3.56, *P* = 0.000), GAD-7 presence of anxiety (OR: 6.40, *P* = 0.001), PHQ score (odds ratio: 2.50), and diagnosis of ED on the IIEF-5 scale (OR: 7.50, *P* = 0.001) were significantly associated with Sexual Health relationship stress and Sexual health satisfaction (Tables 2, 3).

**Table 2:** Correlation of PHQ score, GAD score, and ISS score to covid outbreak

	Sexual satisfaction post covid				p
	Very strong	Strong	WEAK	No desire	
*PHQ score*					
Minimal or no depression 4	20 (60.6%)	4 (12.1%)	5 (15.2%)	4 (12.1%)	0.017
Mild depression 5–9	45 (64.3%)	3 (4.3%)	15 (21.4%)	7 (10.0%)	
Moderate depression 10–14	17 (63.0%)	6 (22.2%)	2 (7.4%)	2 (7.4%)	
Severe depression 15–19	99 (65.6%)	23 (15.2%)	20 (13.2%)	9 (6.0%)	
*GAD score*					
No anxiety 5	21 (63.6%)	3 (9.1%)	5 (15.2%)	4 (12.1%)	0.07
Mild anxiety 5–9	44 (62.9%)	5 (7.1%)	13 (18.6%)	8 (11.1%)	
Moderate anxiety 10–14	18 (66.7%)	5 (18.5%)	2 (7.4%)	2 (7.4%)	
Sever anxiety 15	107(70.9%)	16 (10.6%)	19 (12.6%)	9 (6.0%)	
*ISS score*					
No stress 30	21 (63.6%)	45 (64.3%)	17 (63.0%)	106 (70.2%)	0.27
Stress 30	4 (12.1%)	13 (18.6%)	8 (29.6%)	29 (18.5%)	
Sever stress 70	8 (24.2%)	12 (17.1%)	2 (7.4%)	17 (11.3%)

**Table 3:** Linear regression analysis

	Beta	t	Sig	95.0% confidence interval for B	
				Lower Bound	Upper Bound
(Constant)		8.862	.000	.287	.451
PHQ_score	.250	2.503	.013	.039	.326
GAD_score	.596	6.482	.000	.301	.563
financial_problem_lockdown	.124	3.569	.000	.082	.283
smoking_precovid	− .006	− .275	.783	− .076	.057
smoking_postcovid	.034	1.555	.121	− .013	.115
sports_practice	− .002	− .080	.936	− .082	.076
PDE_precovid	− .013	− .534	.594	− .093	.053
PDE_post_covid	.015	.657	.512	− .046	.093

## Discussion

Our government banned schools and institutions, flights into and out, as well as all public and private transportation, during curfew hours, following a dramatic increase in the number of detected cases in mid-March 2020. To combat the development of COVID-19, all sports were halted and many social activities were outlawed [14].

Sexual health, according to the World Health Organization, is "not just the absence of disease, dysfunction, or disability." Sexual health necessitates a positive and respectful attitude toward sexuality and Sexual Health relationships, as well as the ability to have joyful and safe Sexual Health encounters that are free of compulsion, prejudice, and violence." [2] It has been connected to a number of variables, including mental stress, job hours, pregnancy, and so on. A well-known indication of Sexual health and well-being is Sexual health satisfaction [15].

It is well known that the differences in how males and females respond to stress may have an impact on their Sexual health satisfaction [16]. Males with Sexual health dysfunction or a recognizable Sexual health condition are regarded inferior by their lovers and friends in conservative eastern countries. Male Sexual health function is equated with masculinity, and any expressed displeasure is thought to jeopardize manly genital pride and male supremacy [17].

The majority of the participants, in our study as well as other studies, were between the ages of 25 and 45, which corresponds to the average age of peak Sexual health activity in eastern societies [7, 18].

Most of the time, this unequal Sexual health satisfaction between males and females is attributed to the couples' lack of understanding on how to cope sexually with their partner, which is primarily due to misunderstanding [7].

Based on PHQ-9 scores and GA-7, more than half of our participants significantly didn’t have depressive disorder and anxiety, respectively. The same finding was noted by a national study [7] as well as an international study [19].

Financial issues (*P* = 0.000), smoking habits prior to lockdown (*P* = 0.001), spots practice (*P* = 0.001), smoking during lockdown (*P* = 0.001), presence of depressive disorder on the PHQ-9 total score (*P* = 0.001), diagnosis of anxiety on the GAD-7 score (*P* = 0.001), and presence of ED on the IIEf-5 questionnaire (*P* = 0.001) were all significantly related to the ISS total score.

Similarly, the total ISS score of males was shown to be substantially related to their age, education level, and occupation (clerks and manual workers) [7]. Moreover, lower educational levels are linked to male Sexual health dysfunction [20]. On the other hand, male Sexual health dysfunction is also a risk factor for Sexual health stress since it prevents men from having satisfying Sexual health relationships [7, 21].

Neither participants age nor marriage duration were significantly associated with Sexual health stress. On the contrary, husband's age of more than 35 years and a marriage length of 5–10 years were both associated with Sexual health stress. The rational for their significance was that spouse becomes accustomed to the situation, accepts it, and is less inclined to seek help [7, 22, 23].

On multivariate analysis, the results of a linear regression analysis of factors affecting ISS score in males revealed that financial issues (odds ratio [OR]: 3.56, *P* = 0.000), GAD-7 presence of anxiety (OR: 6.40, *P* = 0.001), PHQ score (odds ratio: 2.50), and diagnosis of ED on the IIEF-5 scale (OR: 7.50, *P* = 0.001) were significantly associated with Sexual health relationship stress and Sexual health satisfaction.

Our findings suggest that Sexual health stress is a major public health concern, and that emotional issues are likely to contribute to the occurrence of these issues. To confirm the findings and examine changes over time, long-term follow-up of changes in Sexual health function and satisfaction after the pandemic is recommended. We need to raise societal awareness of the need of acknowledging such complaints in order to remove the stigma associated with them.

The main strength of our study was a multicenter study, alleviating the bias in population culture and providing diverse aspects of the studied condition. The main limitations for our study was the small sample size. But, this is attributed to poor collaboration of the participants and their eager to share such research projects. Moreover, self-reporting of satisfaction prior to the lockout was used in this study, which is prone to recall and desirability bias. Prior to the COVID-19 pandemic, we did not have IIEF-5 scores, thus comparisons with baseline levels were impossible.

## Conclusion

During and after COVID-19lockdown, the presence of anxiety on the GAD-7 scale, the PHQ score, and the diagnosis of ED on the IIEF-5 scale were all independent predictors of Sexual health. Intervention approaches are offered to help those who have been impacted, particularly after the pandemic.

## Data Availability

The datasets used and/or analyzed during the current study available from the corresponding author on reasonable request.

## Abbreviations

ED: Erectile dysfunction
GAD-7: Generalized anxiety disorder-7 questionnaire
IIEF-5: International index of erectile function questionnaire
PHQ-9: Patient health questionnaire
